# Cultural perspective on religion, spirituality and mental health

**DOI:** 10.3389/fpsyg.2025.1568861

**Published:** 2025-04-02

**Authors:** Angie Cucchi, M. Walid Qoronfleh

**Affiliations:** ^1^School of Social Sciences and Professions, London Metropolitan University, London, United Kingdom; ^2^Healthcare Research & Policy Division, Q3 Research Institute (QRI), Ann Arbor, MI, United States

**Keywords:** mental health, cross cultural, spirituality, religion, culture, intersectionality, anti-colonialism, decolonization

## Abstract

Over the past decade, spirituality and religiosity have gained increasing recognition in the field of mental health, with more individualized approaches emerging. Many mental health professionals have begun integrating aspects of religion and spirituality into their practice and modern psychological therapies have also incorporated principles from ancient Eastern traditions and various worldwide religions. However, these integrations have remained at surface level, assimilating concepts and practices that have been stripped of their ontological framework. The intersection between culture, spirituality and mental wellbeing remains largely underexplored. This can contribute to misunderstandings regarding the conceptualization of mental illness across different cultures and may result in the tokenistic application of ‘*culturally sensitive’* interventions, which can perpetuate the disillusionment that some individuals may experience towards mental health services. This cultural perspective paper examines the intersection between culture, religious/spiritual beliefs and mental health. It engages with some of the concepts described above and embodies an anti-colonialist stance, demonstrating the authors’ commitments toward the decolonization of the field of mental health.

## Introduction

The search for meaning that extends beyond one’s own life and gives sense to human existence and suffering transcends temporal and geographical boundaries. History is ripe of anecdotes and more scholarly examples that testify how, since the dawn of time, human beings have attempted to find answers to the existential questions that are inherently connected to being alive. *After all, if there is a meaning in life at all, then there must be a meaning in suffering* (Frankl, 1962, p.9). Spirituality and religion have often been employed as tools to give meaning to existence and hardship and to explain and alleviate physical and emotional suffering (Sharma et al., 2009). Indeed, Scheler (1921) argued that human beings are naturally driven to seek trascendental meaning, a claim that finds support by the fact that throughout history, every civilization and culture have developed their unique frameworks to explain and tolerate life’s inescapable pain and illness. Despite the ultimate shared goal and a certain degree of commonalitites, these frameworks have been undeniably shaped by cultural, geographical, and social factors and have varied greatly, ranging from personal and unstructured journeys where individuals seek a connection with a higher power or purpose—aligning more with spirituality (Joseph et al., 2017)—to organized dogmas and rituals that are characteristic of institutional teleological orthodoxy^1^.

Historically, mental health has also often been linked to religion and spirituality (R/S), with explanatory frameworks for poor mental health frequently residing in the realm of the supernatural. However, the rise and ultimate supremacy of the Enlightment paradigm in the West, which emphasized rationality and empiricism, led to the modernist dominance of science over other disciplines. Science and religion/spirituality started being portrayed as enemies, a move that influenced the emerging fields of psychiatry and psychology, which aimed to explain and alleviate mental suffering through rational means. As a result, religious beliefs and spirituality were marginalized, often deemed unscientific and irrational (Shah, 2022; Sharma et al., 2009). Consequently, their practice was discouraged and frequently disparaged (Cucchi, 2022; Lucchetti et al., 2021).

The conflict between mental health disciplines and religion/spirituality persisted throughout much of the twentieth century (Cucchi, 2022) due to the inherent incompatibility between the principles of postmodernism and those of traditional religious beliefs (Weber and Pargament, 2014). While institutional teleological orthodoxy depends on the idea of a single reality and an absolute Truth that can be communicated through language, postmodernist thought suggests that there are as many realities as there are individuals, as these realities are co-constructed through interpersonal interactions. In this view, language is seen as a tool for creating reality, rather than merely a means of communicating it. This, according to Shah (2022) further continued to banish religion and spirituality from the academic debate on mental health.

Historically, religion and science maintained a complementary relationship, though this dynamic shifted with the rise of secularization, creating a perceived and continuous divide between the two. The secularization of knowledge, however, is primarily a Eurocentric movement (Berger and Luckmann, 1969) evolving during the renaissance age, while non-Western cultures generally embrace more holistic ontological and epistemological perspectives. For instance, the Islamic worldview has always considered religion and science to be complementary as evidenced during the period of the Golden Age of Islam (Al-Nuaimi et al., 2020). Fernando (2014) explains how in many Eastern cultures, meaning and a sense of purpose are often derived from the interconnectedness between the individual, the community, and the broader environment, including the metaphysical realm. Consequently, well-being, including mental well-being, is understood as a balance among physical, mental, social, and spiritual elements. Given this interconnectedness lens, explanatory models (Kleinman, 1977), help-seeking behaviors, and healing practices can be significantly different from those found in the West.

In recent decades, there has been a growing recognition of the significance of culture in shaping ontological and epistemological frameworks. This recognition, along with the practical implications that different ontological and epistemological positions can have on daily life, has prompted mental health governing bodies to encourage professionals to consider patients’ cultural backgrounds when providing care. Spiritual and religious beliefs are often integral to an individual’s culture, with more than 80% of the world’s population identifying with an insitutional orthodoxy and many others describing themselves as ‘spiritual’ (Moreira-Almeida et al., 2021). Consequently, it has been argued that patients’ spiritual and religious beliefs should be incorporated into a holistic approach to care and wellbeing (American Psychological Association, 2008; Moreira-Almeida et al., 2016; World Health Organization, 1998), when appropriate. Furthermore, the importance of training and education for mental health professionals in addressing spirituality and religion has been acknowledged (American Psychological Association, 2008; Moreira-Almeida et al., 2016).

Several psychological and sociological theories explore the relationship between culture, religious/spiritual beliefs, and mental health. Amongst these theories, the existential one stands out in its relevance to this perspective paper. Prominent psychologists have suggested that existential anxiety- a profound feeling that arises when one confronts the inevitable reality of life’s impermanence, existential isolation, and the meaningness of the human experience- is an instrinsic aspect of being human, (Frankl, 1962; May, 1969; Yalom, 1980). Therefore, existential anxiety is believed to be the ordinary state for humans. Instead, when individuals resort to cognitive avoidance, repression, or fail to appropriately process these fundamental existential truths, they may experience more significant mental health difficulties (Frankl, 1962; May, 1969; Yalom, 1980). It is precisely this existential angst and the awareness of the meaningness of the human experience that has been argued to be a catalyst for individuals to seek existential meaning through religious and/or spiritual beliefs (Frankl, 1962; May, 1969; Yalom, 1980).

R/S become vital tools to navigate existential fears and the uncertainty of the human condition, providing a sense of purpose, meaning and connection (May, 1969). However, May also noted that some cultures tend to avoid existential anxiety by disengaging from deeper reflections on life, instead emphasizing personal acheivement and success. However, this process can exacerbate feelings of isolation and alientation that are intrinsic to the human experience. In fact, the glorification of personal achievement and material success can steer individuals away from fostering connections, inner growth and embracing communal values, leaving them trapped in a cycle of superficial achievements that fail to provide deep meaning and inner acceptance. May further noticed that this cultural tendency often relegates illness, frailty, and death to secluded spaces in an attempt to create a misleading sense of security and certainty.

The often-stark demarcation between religion and science in the European countries and North America was particularly notable during the 20th century. Instead, in recent years a shift toward greater acceptance of religion, particularly of spirituality within the scientific community is becoming apparent. An intriguing emerging field of research termed ‘*neurotheology*’, exemplifies this shift. Neurotheology offers new insights into the connections between religion, health, and the human experience. By crossing traditional scientific boundaries, it integrates disciplines such as religion, theology, philosophy, and anthropology with neuroscience fields like cognitive science and psychology. Neuroimaging studies have revealed distinct changes in brain regions during prayer and meditation practices. Interestingly, this investigation has also discovered distinctions in brain activity between secular forms of meditation and those rooted in religious traditions. This interdisciplinary approach has already yielded valuable insights, enhancing our understanding of the well-documented links between religiosity and health (Al-Nuaimi et al., 2020).

A renewed interest in the topic has emerged following evidence-base that shows that spiritual/religious beliefs have an impact on mental health, that R/S practices are often used as coping strategies and that addressing the above can have a positive impact on health-related outcomes (Moreira-Almeida et al., 2021). At the same time, the role of culture and the intersectionality between this, mental health and R/S beliefs needs to be contemplated.

## Intersection and intersectionality: culture, mental health and religious/spiritual beliefs

Crenshaw (1989) warns that any exploration that does not address intersectionality continues to perpetrate a superficial approach to an antidiscriminatory doctrine. Her use of the term as a lens that sheds light on power hierarchies and power interlocks and intersects (Crenshaw, 2020) seems appropriate to capture the complexity and the depth of the relationship between the above terms, as described in the previous section. Power dynamics have often been disregarded or implicitly examined in mental health research. Instead, this perspective article aims to address them explicitly. After all, an individual’s mental health, including its expression and perception, is influenced by a range of intertwined cultural, socio-economic, historical and political factors, as well as characteristics such as ethnicity, class, gender, lifestyle, genetic predisposition, etc.…

The literature highlights several ways in which culture, mental health and R/S intersect both at a micro and macro level. On a micro level, an individual’s cultural background influences explanatory models of mental health, with non-Western cultures favouring more holistic and spiritual explanatory models (Fernando, 2010). This can significantly impact help-seeking behaviors, expectations regarding treatment, and overall engagement. At the same time, culture also shapes the presentation and the expression of psychological distress, both at a micro and macro level. For instance, while the core symptoms of depression and anxiety are universally recognized (Kirmayer, 2001), there is significant evidence that non-Western cultures tend to favour somatic metaphors over psychological ones when expressing psychological distress associated with these conditions (Al Adawi et al., 2011).

Furthermore, the interplay of culture, mental health and R/S in the manifestation of the phenomenological characteristics of other psychiatric presentations is even more compelling. Empirical data confirms that the content of obsessive-compulsive disorder (OCD) obsessions is shaped by cultural concerns, with more religious obsessions found in the Middle East, whereas more aggressive ones more prevalent in South America (Fontenelle et al., 2004; De Bilbao and Giannakopoulos, 2005; Hassan et al., 2024). In East Asia it is common for indivduals to experience more symmetry and order-related obsessions, whereas more superstitious ones are present in the African culture (Hassan et al., 2024). Lastly, the indian subcontinent features more contamination-related obsessions whereas the West culture more checking and counting ones (Hassan et al., 2024).

A similar trend is seen in the phenomenology of psychosis, with more religious and mystical themes found in hallucinations of Middle Eastern individuals compared to Europeans who instead report more persecutory and hostile ones (Kent and Wahass, 1996). Similarly, the phenomenological characteristics of what is now known as Anorexia Nervosa (AN) have also been shown to be shaped by the intersection between culture and religion (Bell, 2003). In her book titled ‘*Holy Anorexia’*, Bell highlights the link between self-imposed pathological food restriction and religious ascetism by drawing on culturally-specific historical examples of medieval Christian female saints who embodied their religious devotion and spiritual purity by virtue of extreme fasting and self-abnegation. Extreme self-starvation in this cultural context was not only not viewed as a sign of mental illness, but was instead admired and praised to the extent that the young women who died in their quest to master ultimate control over their physical need for food were revered as saints.

The exploration of the historical religious, socio-cultural and gendered aspect of AN, as outlined by Bell (2003), provides a compelling example of the intersection between what are now deemed to be mental health symptoms (extreme food restriction), culture and religion on a macro level. It has been argued that the emphasis on the interconnedness between sanctity, purity and bodily control characteristic of specific cultural and religious frameworks has influenced the ways in which women are expected to experience, relate to and embody their physical being (Bell, 2003). This perspective challenges the traditional biomedical paradigm of AN as a discrete category of mental illness by considering how religious and cultural frameworks can influence not only the development of symptoms, but also the very definition of ‘*mental illness’* and ‘*psychopathology’*. The implications of a diagnostic manual for the classification of psychiatric distress that- until recently- listed ‘*intense fear of gaining weight’* as one of the essential diagnostic criteria (DSM IV-TR- American Psychiatric Association, 2000) were that all the self-starvation cases documented worldwide that did not meet this diagnostic criteria (Al-Adawi et al., 2004; Bell, 2003; Lee et al., 1993; Tareen et al., 2005) were not recognized, deemed ‘atypical’, or overlooked.

Fernando (2014) plays a crucial role in raising awareness of the structural racism that permeates the discipline and underpins the intersectionality between mental health, culture and religion on a macro level. For example, some symptom clusters are considered ‘legitimate’ and ‘valid’ while any deviation from these are labeled as ‘distortions’ and are not given legitimacy. As expected, the costellations of symptoms deemed to be ‘legitimate’ are those detected in Western contexts.

Interestingly, AN was initially conceptualized as a culturally-bound syndrome (Swartz, 1985) due to the fact that the phenomenological characteristics of the ‘*disorder*’, as conceptualized by the psychiatric diagnostic manuals prior to the latest edition, were exclusively found in Western cultures that glorify thinness and specific body types. Any other presentation that did not conform to these criteria was deemed not authentic enough to ‘deserve’ legitimacy from the medical profession. The extent to which structural inequalities pemeate the structure of knowledge is evidenced by the advocacy work to update the diagnostic criteria of classification manuals to recognize cultural variations to the phenomenology of AN (Becker et al., 2009). Some scholars have argued that AN is better understood as a culturally-reactive syndrome (DiNicola, 1992; Keel and Klump, 2003), a condition that develops and is shaped by the specific socio-cultural context, including the religious one.

Another example worth considering to reflect on the intersection between mental health, culture and religion/spirituality is the practice of self-flagellation performed by Shia Muslims during Ashura, which occurs annually on the 10th of Muharram, the first month of the Islamic calendar, to commemorate the death of the grandson of the prophet Mohammed. The political, religious and ethical implications, including the legitimacy of this practice are beyond the scope of this paper and will not be explored here. Instead, the authors would like to draw the readers’ attention to the relationship between medically recognised mental health symptoms, culture and religion, as discussed above. Self-flagellation is generally deemed to be a significant sign of poor mental health and is associated with a wide range of psychiatric presentations (Sadath et al., 2023). Yet, in the context of culturally-specific religious practices, positive elements have been identified (Hobson et al., 2017; Whitehouse, 2018; Xygalatas 2022). The authors describe the value of self-flagellation and other forms of self-inflicted physical pain in the context of religious ceremonies and practices. The value being emotional catharsis, collective psychological support in the face of historical grief and the experience of oppression, as well as fostering a sense of belonging and solidarity which increases wellbeing and spiritual healing.

Watters (2010) warned of the dangers of the ‘*globalization’ of the American psyche’* and the imposition of Western psychiatric diagnosis on indigenous cultures. He highlighted the impact of the aggressive campaigns of pharmaceutical companies that led to the reconceptualisation of sadness in Japan, as well as the export of the concept of PTSD and the Western ideals of beauty, respectively, in post-tsunami Sri-Lanka and Hong Kong. Furthermore, Watters stressed the need to understand the cultural factors underpinning schizophrenia. The book highlights the importance of avoiding a draconian application of Western standards of wellbeing and ensuring that the classification of phenomena as normal or abnormal relies on a thorough understanding of the micro and macro factors that underpin the relationship between culture, mental health and R/S.

Although it is undeniable that the most recent classification manuals have incorporated cultural sensitivity in their approach to psychiatric distress, it has been argued that the influence of culture and R/S on mental health is not uniform, but it varies based on the social location and hierarchy that the individual inhabits. These factors shape the messages individuals receive (Van Mens-Verhulst and Radtke, 2008) and influence how social inequalities impact mental health and wellbeing (Rosenfield et al., 2006). Within the same cultural or religious background, socio-economic status (SES), gender, education, ethnicity, geographical location, etc.… mediate the relationship with psychological wellbeing.

This perspective paper takes a further step to argue that the same concept of mental health and psychopathology requires a critical evaluation that takes into consideration structural power and hierarchies.

## Current and future trends: the way forward

In recent years there has been a significant increase in psychotherapeutic modalities that have incorporated spiritual and religious elements (Kahle and Robbins, 2004). Historical models such as Transpersonal Psychology, existentialism, Jungian and Franklin’s work will not be discussed here. The reader is referred to Cucchi (2022), Frankl (1962), Tart (1975), and Jung (1973) for further details. The modality that has attracted most attention and has been more formally adapted to incorporate religious (de Abreu Costa and Moreira-Almeida, 2022) and spiritual beliefs (Correa and Sandage, 2018) is Cognitive Behavioural Therapy (CBT). In addition to generic adaptations, the model has also been integrated with more specific values from different religious beliefs-system such as Christianity (Collins, 2014), Islam (Cucchi, 2022), as well as Eastern spiritual practices (Hayes et al., 2004; Kabat-Zinn, 1994).

Religious-based CBT is deeply entrenched in the relevant institutional teleological orthodoxy and aims to align a person’s belief system with the religious teachings. It employs prayers and other religious practices as behavioral activation, as well as the reading of and the use of sacred books for cognitive restructuring (Cucchi, 2022; de Abreu Costa and Moreira-Almeida, 2022). Instead, spiritually modified CBT is rooted in Eastern philosophical principles and seeks to foster self-awareness and existential growth. It relies on practices such as acceptance and meditation to achieve these goals (Correa and Sandage, 2018). Specific modalities such as Mindfulness-based Stress Reduction (Kabat-Zinn, 1990), Mindfulness-based Cognitive Therapy (Segal et al., 2018), Acceptance and Commitment Therapy (Hayes et al., 2004) and Compassionate Mind (Gilbert, 2010) have been developed and many more modalities integrate spiritual beliefs.

However, all of the aforementioned approaches use assimilative integration to incorporate spiritual/religious beliefs into a mainstream Western psychotherapeutic model. This raises significant ontological and epistemological dilemmas, as discussed specifically in the context of the integration of Islamic values into CBT in Cucchi (2022). Most Western models have their roots in individualism, self-actualisation and a dichotomy between the mind and the body (Cushman, 1995). They also depend on Western-standards of prescribed emotional expression, as well as biopsychological explanatory models and treatments that primarily rely on language, or medications. The latter two are carefully monitored through evidence-base practices. However, as previously discussed, these beliefs and ways of working often do not resonate with individuals from collectivist cultures whose ontological and epistemological positions tend to be significantly different.

Adopting a critical perspective implies examining the existing distribution of power, social hierarchies and systemic biases. It involves reflecting on the neocolonial dangers associated with assimilative integration in the current socio-economic and political context, particularly when incorporating cultural and spiritual beliefs and practices that are ontologically opposed to the dominant mainstream model. Marsella (2012) condemns this process as a solidification of cultural hegemony and Fernando (2014, p.81) describes it as a ‘*marketing ploy’.* He warns that this practice would contribute to the disillusionment of people from non-Western cultures, whose conceptualisation of health and collective human experience is very different. Fernando also cautions professionals that assimilative integration would demean the meaning of R/S as conceptualized in most cultures.

In many non-Western cultures, religion and spirituality are not simply an ‘add-on’. Although it is acknowledged that spiritual beliefs can vary significantly even within the same cultural heritage depending on regions and tribes, some universal elements can be identified. For example, many indigenous cultures believe in an indissoluble spiritual connection between the cosmos, nature and human beings, asserting that all living and non-living things posess a spirit (Johnson and Kraft, 2017). A similar view is held in many African cultures, where the conceptualisation of the world is deeply rooted in the cosmic and ancestral connection between the spiritual and the material (Mbiti, 1990). Hinduism also rejects the concept of dualism. Instead, it asserts that all living things possess an Atman (a soul) with inanimate objects believed to be the expression of a divine presence (Chakravarti, 2010). In the Chinese culture, this profound, dynamic and balanced connection is simbolised by the concept of the ‘Tao’ (Kirkland, 2004).

It can be argued that integrating spirituality into Western models that have such different ontological basis, reinforces neocolonialist practices such as cultural appropriation and imperialism. Although important steps have been made in our understanding of the intersectionality of mental health, culture, and religion/ spirituality, it is crucial for professionals to build on this understanding through an anti-discriminatory and anti-racist stance. This implies a recognition that the current definition of mental health carries significant imperialistic and hence, racist, connotations. Therefore, moving away from a superficial and tokenistic integration of concepts into mainstream approaches, professionals should commit to the decolonization of the field of mental health by reconceptualizing the very same definition of mental health and mental illness.

Fernando (2014) calls for a flexible and dynamic reconceptualization in which social, spiritual and cosmological forces, as well as genetic and psychological ones are contemplated. The present authors also call for the incorporation of political and economic influences. Such approach would acknowledge that the explanatory models of mental illness vary according to the cultural background, with none being superior to the others. As such, approaches such as African, Buddhist, Aboriginal and Islamic Psychotherapy should be routinely offered to our clients worldwide, in addition to Western-based models. Fernando (p. 175) describes this as ‘*diversity within unity and cultural relativism within a unified approach’*, a concept that remind us of the di-unital perspective found in African Psychology (Matthews and Nichols, 2022). Reevaluating the concept of mental illness, recognizing the equal value of all approaches and ensuring real inclusion through the decolonization and the diversification of the field of mental health would lead to the development of a very different discipline, one that is free from the legacy of colonialism and, hence, fairer and more inclusive for all.

## Conclusion

The interplay between religion, spirituality, and mental health is deeply influenced by cultural perspectives. Different societies and traditions shape how individuals experience, interpret, and cope with mental health challenges through spiritual and religious frameworks. For many, faith provides a source of meaning, resilience, and social support, offering comfort in times of distress. Religious and spiritual practices, such as prayer, meditation, and ritual observance, can promote psychological well-being and foster a sense of connectedness. However, cultural variations also highlight challenges, such as different explanatory models surrounding mental illness, religious interpretations that may hinder professional treatment, or conflicts between spiritual beliefs and mental health interventions.

A culturally sensitive approach to mental health care has recently been adopted. This acknowledges the significance of religious and spiritual beliefs while integrating evidence-based therapeutic practices. At the same time, this integration has primarily happened by assimilating ideas and practices that have been removed from their original ontological framework into mainstream Western models, reinforcing cultural hegemony and raising concerns about cultural appropriation. Mental health professionals can surely benefit from understanding the cultural and religious backgrounds of their clients, fostering a more holistic approach to treatment. At the same time, this understanding should happen at a deeper level, respecting different cultures, ontological and epistemological positions. This would ensure an anti-discriminatory and antiracist practice. By bridging the gap between spirituality and psychology, societies can create inclusive, compassionate frameworks that support mental well-being while respecting diverse worldviews. Ultimately, recognizing the cultural dimensions of religion, spirituality, and mental health can help cultivate a more inclusive approach to healing.

## Data Availability

The original contributions presented in the study are included in the article/supplementary material, further inquiries can be directed to the corresponding author.

## Footnotes

^1^The authors acknowledge that religion/spirituality (R/S) have roots and encompass different symbolic and theoretical constructs. Exploring these differences is, however, beyond the scope of this paper. For the purpose of this article, the authors use the term (R/S) in a broad, inclusive way that reflect this diversity while maintaining a focus on the shared elements of these constructs.
